# Structural insight into the Ragulator complex which anchors mTORC1 to the lysosomal membrane

**DOI:** 10.1038/celldisc.2017.49

**Published:** 2017-12-26

**Authors:** Zongkai Mu, Lei Wang, Wei Deng, Jiawei Wang, Geng Wu

**Affiliations:** 1 State Key Laboratory of Microbial Metabolism, School of Life Sciences and Biotechnology, Shanghai Jiao Tong University, Shanghai, China; 2 National Center for Protein Science Shanghai, Shanghai, China; 3 State Key Laboratory of Membrane Biology, School of Life Sciences, Tsinghua University, Beijing, China

**Keywords:** mTORC1 signaling pathway, Ragulator, Rag GTPase complex, Crystal structure, EGO-TC, Lamtor

## Abstract

The mechanistic target of rapamycin (mTOR) signal-transduction pathway plays a key role in regulating many aspects of metabolic processes. The central player of the mTOR signaling pathway, mTOR complex 1 (mTORC1), is recruited by the pentameric Ragulator complex and the heterodimeric Rag GTPase complex to the lysosomal membrane and thereafter activated. Here, we determined the crystal structure of the human Ragulator complex, which shows that Lamtor1 possesses a belt-like shape and wraps the other four subunits around. Extensive hydrophobic interactions occur between Lamtor1 and the Lamtor2-Lamtor3, Lamtor4-Lamtor5 roadblock domain protein pairs, while there is no substantial contact between Lamtor2-Lamtor3 and Lamtor4-Lamtor5 subcomplexes. Interestingly, an α-helix from Lamtor1 occupies each of the positions on Lamtor4 and Lamtor5 equivalent to the α3-helices of Lamtor2 and Lamtor3, thus stabilizing Lamtor4 and Lamtor5. Structural comparison between Ragulator and the yeast Ego1-Ego2-Ego3 ternary complex (Ego-TC) reveals that Ego-TC only corresponds to half of the Ragulator complex. Coupling with the fact that in the Ego-TC structure, Ego2 and Ego3 are lone roadblock domain proteins without another roadblock domain protein pairing with them, we suggest that additional components of the yeast Ego complex might exist.

## Introduction

The mechanistic target of rapamycin (mTOR) signal-transduction pathway regulates many aspects of metabolic processes such as protein translation, lipid synthesis and autophagy in a diverse variety of organisms from yeast to human. Deregulation of the mTOR signaling pathway underlies many human diseases including cancer and diabetes [1–4]. The activation of mTORC1, the central player of the mTOR signaling pathway, which consists of mTOR, Raptor and mLST8, is subject to stringent regulation by the availability of nutrients such as amino acids [5–7]. When amino acids are abundant, mTORC1 is translocated to the lysosomal membrane, through the mediation of the heterodimeric Rag GTPases, which consists of RagA or RagB in complex with RagC or RagD [8, 9] and the pentameric Ragulator complex [10, 11]. At the lysosome, the kinase activity of mTORC1 is activated by the small GTPase Rheb, which is also located in lysosomes [12, 13], and then phosphorylates a variety of its target substrates such as eIF4E-binding protein 1 and S6 kinase 1.

The lysosomal membrane-attached Ragulator complex consists of five subunits: Lamtor1, Lamtor2, Lamtor3, Lamtor4 and Lamtor5 (which are also known as p18, p14, MP1, C7orf59 and HBXIP, respectively). The N-terminal region of Lamtor1 contains myristoylation and palmitoylation sites, which anchors the Ragulator complex to the lysosomal membrane [14]. Lamtor2, Lamtor3, Lamtor4 and Lamtor5 all contain roadblock domains [11]. The Rag GTPase complex may interact with the Ragulator complex through the C-terminal domains of RagA/B and RagC/D, which are also roadblock domains [15]. In addition, it was reported that the Ragulator complex serves as a guanine nucleotide exchange factor for RagA and RagB, promoting their GTP binding in exchange of GDP.

Most of the key players of human mTOR signaling pathway are conserved in yeasts. For example, there are two yeast mTOR homologs, TOR1 and TOR2 [16]. The yeast counterparts for human RagA/RagB and RagC/RagD have been identified to be Gtr1 and Gtr2, respectively[17]. In addition, three yeast proteins, Ego1, Ego2 and Ego3, have been found to interact with each other and might function as components of a Ragulator-like complex (known as Ego-TC presently) in yeasts [18–20]. However, given the fact that human Ragulator is a pentameric complex, whereas yeast Ego-TC is only a ternary complex, it is not yet clear all the components of yeast Ragulator-like complex have been identified.

Although the crystal structures of the human Lamtor2-Lamtor3 complex [21, 22] and the yeast Gtr1-Gtr2 complex [15, 23] have been reported, the structural assembly of the holo-Ragulator complex still remains elusive. In this work, we determined the crystal structures of both the human Lamtor4-Lamtor5 complex and the human pentameric Ragulator complex. Our structure shows that Lamtor1 exhibits a belt-like structure and wraps around Lamtor2, Lamtor3, Lamtor4 and Lamtor5. There exist extensive interactions between Lamtor1 and all the other four subunits. Lamtor4 associates with Lamtor5 through β-sheet formation and hydrophobic interactions, similar to the association between Lamtor2 and Lamtor3. Interestingly, both Lamtor4 and Lamtor5 lack the α3-helix feature which are present in Lamtor2 and Lamtor3. Assembly of the Ragulator complex brings an α-helix of Lamtor1 to each of the positions on Lamtor4 and Lamtor5 equivalent to the α3-helices of Lamtor2 and Lamtor3, and thus stabilizing Lamtor4 and Lamtor5. In addition, comparison of the structure of the Ragulator complex and that of the previously reported Ego-TC indicates that the three components of Ego-TC, Ego1, Ego2 and Ego3, are equivalent to Lamtor1-α3, Lamtor5 and Lamtor2, in terms of their spatial positions. This suggests that other components of the yeast Ragulator-like complex might exist.

## Results

### Overall structure of the Ragulator complex

We first determined the crystal structure of the human Lamtor4-Lamtor5 complex to 2.03 Å resolution by the molecular replacement method, using the structure of the previously reported Lamtor2-Lamtor3 complex as a searching model (Table 1). With the structures of the Lamtor2-Lamtor3 and the Lamtor4-Lamtor5 complexes as searching models, we then determined the crystal structure of the human pentameric Ragulator complex to 3.01 Å resolution also by the molecular replacement method, with the *R*_work_/*R*_free_ factors being 22.27%/28.33% (Table 1).

There are three Ragulator complexes in the crystallographic asymmetric unit (Supplementary Figure S1). However, the Ragulator complex behaves to be monomeric in solution, as assayed by the size exclusion chromatography-multiangle light scattering method (Supplementary Figure S2), suggesting that trimerization of the Ragulator complexes in crystals might result from crystal packing.

**Figure 1:**
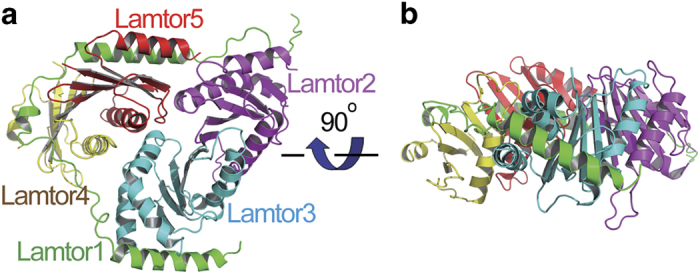
Overall structure of the Ragulator complex. The Lamtor1, Lamtor2, Lamtor3, Lamtor4 and Lamtor5 subunits are colored in green, magenta, cyan, yellow and red, respectively. Each successive panel (**a**, **b**) is rotated as indicated.

In the crystal structure, Lamtor1 displays a belt-like shape, and wraps the other four subunits around (Figure 1). Lamtor2 and Lamtor3 form a semi-independent subcomplex, and so are Lamtor4 and Lamtor5. There is little contact between Lamtor2-Lamtor3 and Lamtor4-Lamtor5 subcomplexes, while there are extensive interactions between Lamtor1 and both.

### Structure of Lamtor1

**Figure 2:**
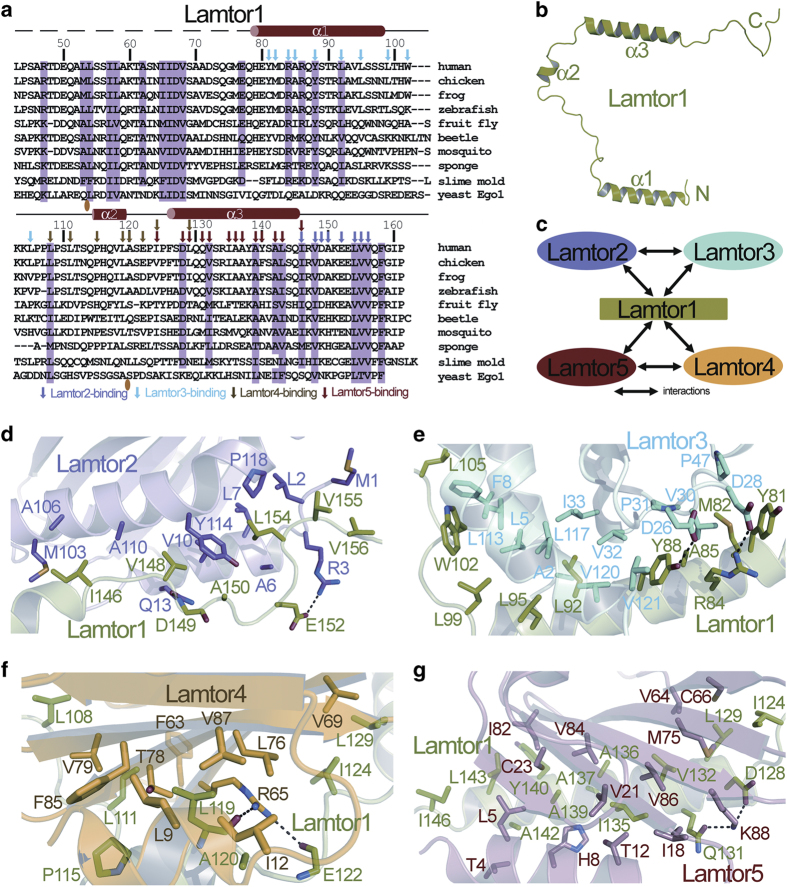
Lamtor1 does not have a stably folded structure on its own, but adopts an extended conformation and enwraps the other four subunits around. (**a**) Structure-based sequence alignment of Lamtor1. Secondary structure elements and residue numbers are indicated above the sequence. Conserved residues of Lamtor1 are shaded in pink. Residues of Lamtor1 interacting with Lamtor2, Lamtor3, Lamtor4 and Lamtor5 are indicated by magenta, cyan, brown and red arrows, respectively. The places that contain loop insertions are marked with orange ovals. (**b**) Lamtor1 adopts an extended conformation and does not have a hydrophobic core on its own. (**c**) Interactions among the five subunits of the Ragulator complex. Interactions are represented as double-sided arrows. (**d**) Interaction interface between Lamtor1 (colored in green) and Lamtor2 (colored in magenta). (**e**) Interaction interface between Lamtor1 (colored in green) and Lamtor3 (colored in cyan). (**f**) Interaction interface between Lamtor1 (colored in green) and Lamtor4 (colored in yellow). (**g**) Interaction interface between Lamtor1 (colored in green) and Lamtor5 (colored in salmon). Hydrogen bonds are indicated as black dashed lines.

The N-terminal region of Lamtor1 serves as a lipidation site for attaching to the lysosomal membrane and does not participate in the assembly of the Ragulator complex, so we did not include it in our Lamtor1 construct for coexpression with the other four subunits. Although we used Lamtor1 (residues 41–161) for assembling the Ragulator complex and for crystallization, only residues 78–161 can be seen in the crystal structure. Residues 41–77 of Lamtor1 are possibly disordered in the crystals, and thus do not contribute clearly observable electron density. Lamtor1 contains three α-helices, but possesses neither tertiary structure nor hydrophobic core on its own (Figure 2a and b). This provides an explanation for our difficulty of expressing and purifying good-behaving monomeric Lamtor1 protein by itself (data not shown).

### Interactions between Lamtor1 and the other four subunits of Ragulator

Lamtor1 interacts with all the other four subunits, and is the major contributor of holding up the Ragulator complex together (Figure 2c). It uses its C-terminal tail to bind to Lamtor2 (Figure 1a and Supplementary Figure S3), uses its α1-helix to associate with Lamtor3 (Figure 1a and Supplementary Figure S4), interacts with Lamtor4 using its α2-helix (Figure 1a and Supplementary Figure S5) and recognizes Lamtor5 through the mediation of its α3-helix (Figure 1a and Supplementary Figure S6).

At the Lamtor1-Lamtor2 interface, I146, V148, L154, V155 and V156 of Lamtor1 pack with M1, L2, A6, L7, V10, M103, A106, A110, Y114 and P118 of Lamtor2 through hydrophobic interactions. Besides, the side chain of Lamtor1-E152 forms a salt bridge with that of Lamtor2-R3, and the main chain of Lamtor1-D149 makes hydrogen bond with the side chain of Lamtor2-Q13 (Figure 2a, d and Supplementary Figure S7).

At the interface between Lamtor1 and Lamtor3, Lamtor1 residues Y81, M82, A85, Y88, L92, L95, L99, W102 and L105 at the α1-helix or the loop immediately following α1 make hydrophobic interactions with Lamtor3 residues A2, L5, F8, V30, P31, V32, I33, L113, L117, V120 and V121. In addition, there is electrostatic interaction between Lamtor1-R84 and Lamtor3-D28, and a hydrogen bond between Lamtor1-Y88 and Lamtor3-D26 (Figure 2a, e and Supplementary Figure S8).

For the Lamtor1-Lamtor4 contacting interface, there exist hydrophobic interactions between Lamtor1-L108, -L111, -P115, -L119, -A120, -I124, -L129 and Lamtor4-L9, -I12, -F63, -V69, -L76, -T78, -V79, -F85, -V87. Moreover, the main chains of Lamtor1-L119 and -E122 form a couple of hydrogen bonds with the guanidinium group of the side chain of Lamtor4-R65 (Figure 2a, f and Supplementary Figure S9).

At the binding interface between Lamtor1 and Lamtor5, extensive hydrophobic interactions occur between residues I124, L129, V132, I135, A136, A137, A139, Y140, A142, L143 and L146 on the α3-helix of Lamtor1 and residues T4, L5, H8, T12, I18, V21, C23, V64, C66, M75, I82, V84 and V86 on the α1-helix and the β1, β3, β4 and β5 strands of Lamtor5. Meanwhile, the side-chain amide group of Lamtor1-Q131 and the side-chain carboxyl group of Lamtor1-D128 form charge-stabilized hydrogen bonds with the amino group of Lamtor5-K88 side chain (Figure 2a, g and Supplementary Figure S10).

### Lamtor1 stabilizes Lamtor4 and Lamtor5 by filling up the gaps corresponding to the positions of the α3-helices of Lamtor2 and Lamtor3

**Figure 3:**
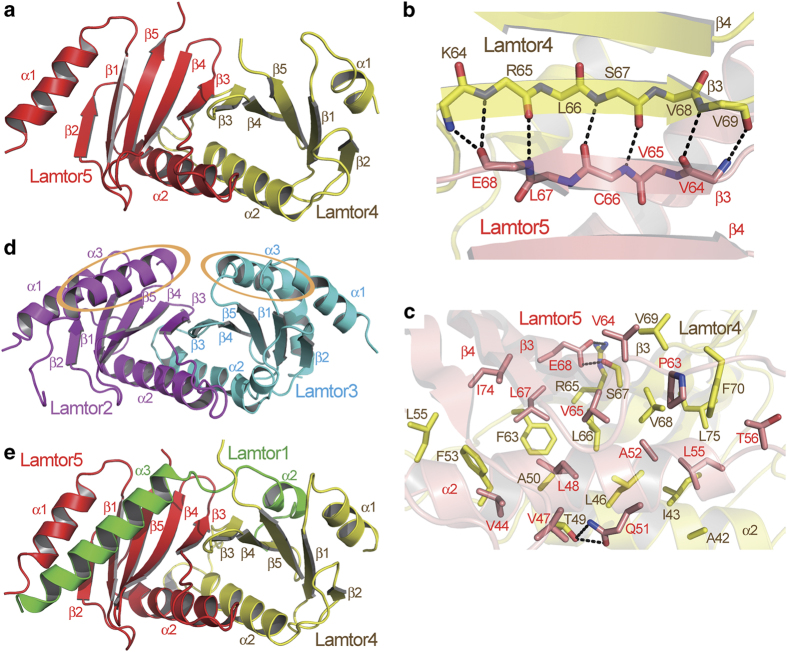
The Lamtor4-Lamtor5 subcomplex is stabilized through association with Lamtor1. (**a**) Structure of the Lamtor4-Lamtor5 subcomplex. (**b**, **c**) Main-chain interactions (**b**) and side-chain interactions (**c**) between Lamtor4 and Lamtor5. Carbon atoms of Lamtor4 and Lamtor5 are colored in yellow and salmon, respectively. Hydrogen bonds are indicated as black dashed lines. (**d**) Structure of the Lamtor2-Lamtor3 subcomplex. Note that both Lamtor4 and Lamtor5 lack the α3-helix features present in Lamtor3 and Lamtor2. (**e**) The α2- and α3-helices of Lamtor1 fill in the gaps on Lamtor4 and Lamtor5, respectively, as compared with the Lamtor2-Lamtor3 complex. Therefore, the Lamtor4-Lamtor5 subcomplex is stabilized.

Within the Ragulator complex, Lamtor4 and Lamtor5 form a subcomplex (Figure 3a), through β-sheet formation between their β3 strands (Figure 3b), as well as extensive hydrophobic interactions between their α2-helices as well as β3 and β4 strands (Figure 3c). The mode of their interaction is very similar to that of the interaction between Lamtor2 and Lamtor3 (Supplementary Figure S11).

Interestingly, both Lamtor4 and Lamtor5 only possess two α-helices, whereas Lamtor2 and Lamtor3 contain three. Comparison between the Lamtor4-Lamtor5 subcomplex and the Lamtor2-Lamtor3 subcomplex shows that Lamtor4 and Lamtor5 lack the feature of the α3-helix, which is present in both Lamtor2 and Lamtor3 (Figure 1a and d, note the orange ovals in Figure 1d). On the other hand, within the Ragulator complex, an α-helix from Lamtor1 fills in each of the gaps on Lamtor4 and Lamtor5 corresponding to the positions of α3-helices of Lamtor2 and Lamtor3: the α2-helix of Lamtor1 fills in the gap on Lamtor4, whereas the α3-helix of Lamtor1 fills in the gap on Lamtor5 (Figure 3d). In this way, the Lamtor4-Lamtor5 subcomplex is stabilized by Lamtor1, and by their assembly into the Ragulator complex.

### The yeast Ego-TC complex only corresponds to half of the human Ragulator complex

**Figure 4:**
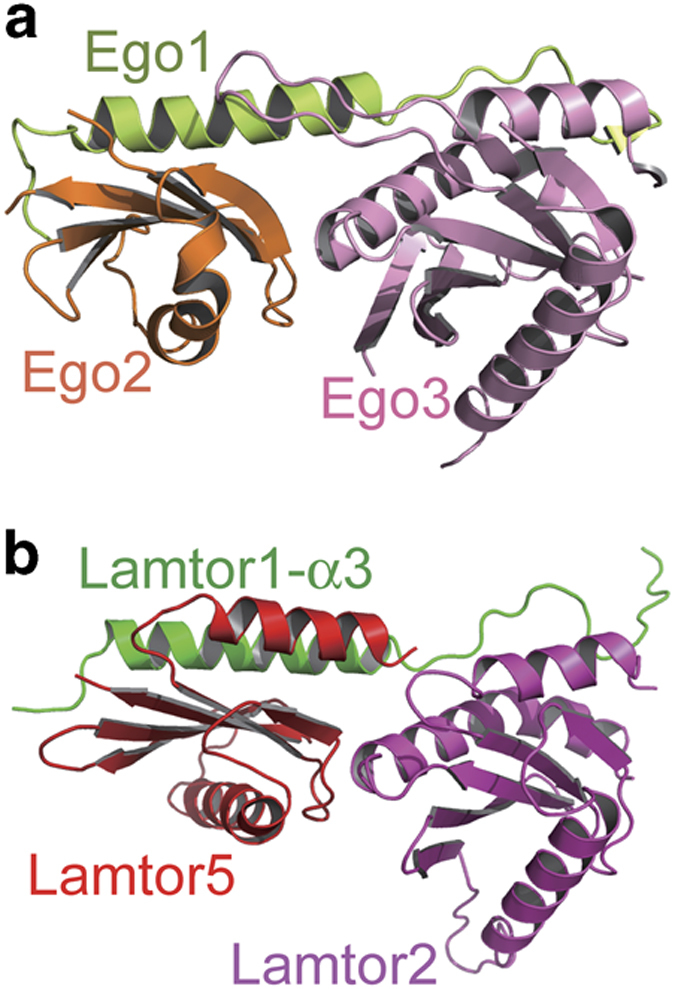
The Ego-TC complex corresponds to half of the Ragulator complex. (**a**) Structure of the previously reported Ego-TC complex. The Ego1, Ego2 and Ego3 subunits are colored in limon, orange and pink, respectively. (**b**) Structure of the Lamtor1-α3-Lamtor5-Lamtor2 subcomplex, which is highly similar in the overall assembly of the Ego-TC complex. Lamtor1-α3, Lamtor5 and Lamtor2 are colored in green, red and magenta, respectively, and their spatial positions correspond to those of Ego1, Ego2 and Ego3, respectively.

The Ego-TC, comprising of Ego1, Ego2 and Ego3, has been identified to be the yeast counterpart of the Ragulator complex [16–18]. In the structure of the Ego-TC complex, Ego1 contains a single long α-helix and a short β strand, and interacts with both Ego2 and Ego3, which are also roadblock domain-containing proteins. On the other hand, there is no contact between Ego2 and Ego3 (Figure 4a). A comparison between the Ego-TC and Ragulator structures reveals that the overall assembly of Ego-TC as well as the spatial position of every one of its subunits is strikingly similar to those of half of the Ragulator complex. Ego1 corresponds to the C-terminal half of Lamtor1 including its α3-helix and the C-terminal tail, Ego2 is equivalent to Lamtor5 and Ego3 is the counterpart of Lamtor2 (Figure 4b). The C-terminal half of Lamtor1 also links Lamtor5 and Lamtor2 together, which do not have direct association with each other. Considering the fact that Ragulator possesses five subunits while Ego-TC only has three, as well as the fact that roadblock domain proteins usually exist in pairs such as the Lamtor2-Lamtor3 pair and the Lamtor4-Lamtor5 pair, it is very probable that additional components of the Ego complex remain to be discovered.

### Mutating or deleting the C-terminal conserved LVVxF motif of Lamtor1 does not affect the interaction between Ragulator and the Rag GTPase complex *in vitro*

**Figure 5:**
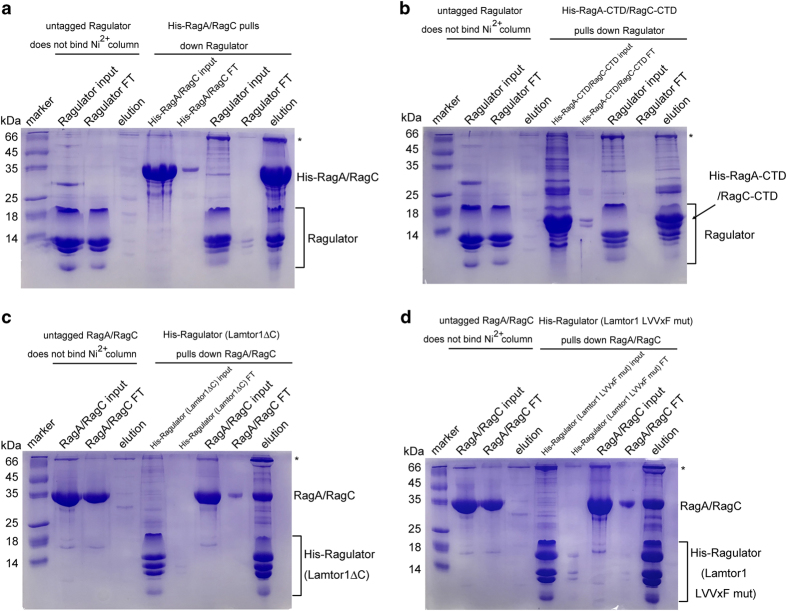
Mutation or deletion of the C-terminal conserved LVVxF motif of Lamtor1 did not affect the association between Ragulator and the RagA-RagC GTPase complex *in vitro*. (**a**) Our purified Ragulator complex interacted with the RagA-RagC complex, as examined by the Ni^2+^-NTA affinity chromatography assay. (**b**) The Ragulator complex also associated with the RagA-CTD-RagC-CTD complex. (**c**) Deletion of residues 151–161 of Lamtor1 did not apparently disrupt the complex formation between Ragulator and the RagA-RagC complex. (**d**) Point mutation of residues L^154^VVQF^158^ to D^154^DDQD^158^ also did not substantially affect the binding between Ragulator and the RagA-RagC complex. The ‘*’ symbol stands for a nonspecific band copurified with our target proteins. FT, flow-through.

One of the major functions of the Ragulator complex is to serve as a lysosome anchor, which recruits the heterodimeric Rag GTPase and its associated mTORC1 complex to the lysosomal surface [10]. The N-terminal region of Lamtor1 contains the lipidation sites [10] and is not supposed to be involved in associating with the Rag GTPase complex. Indeed, purified Ragulator complex with residues (1–40) of Lamtor1 deleted associated with the RagA/RagC complex, as demonstrated through the Ni^2+^-NTA affinity chromatography assay (Figure 5a and Supplementary Figure S13a). The C-terminal domains of both RagA and RagC are roadblock domains, and have been previously suggested to mediate the association with the Ragulator complex [15]. In support of this notion, purified RagA-CTD/RagC-CTD complex interacted with the Ragulator complex (Figure 5b and Supplementary Figure S13a).

Examination of the Ragulator structure shows that the C-terminal part of Lamtor1, especially residues L^154^VVQF^158^, is solvent exposed and is among the most conserved parts of the Ragulator surface (Figure 2a and Supplementary Figure S12). To investigate the functional importance of this motif, we either deleted residues 151–161 of Lamtor1 or mutated L^154^VVQF^158^ to D^154^DDQD^158^, and investigated whether the resulting Lamtor1 mutants could still support the association between Ragulator and the Rag GTPases. To our surprise, deleting or mutating the highly conserved C-terminal region of Lamtor1 did not appreciably affect the Ragulator-Rag complex formation (Figure 5c, d and Supplementary Figure S13b). Presumably, other subunits of the Ragulator complex besides Lamtor1, such as Lamtor2 and Lamtor3, contribute to the binding between Ragulator and the Rag GTPases.

## Discussion

The pentameric Ragulator complex plays a critical role in the mTOR signal-transduction pathway by bringing the mTORC1 complex to the lysosomal surface through the mediation of the Rag GTPases, thus mTORC1 can be activated by the lysosome-located small GTPase Rheb. Our structure of the Ragulator complex reveals that Lamtor1 possesses a belt-like shape and encircles the two subcomplexes, Lamtor2-Lamtor3 and Lamtor4-Lamtor5. Extensive associations, predominantly being hydrophobic interactions, occur between Lamtor1 and every one of the other four subunits. Interestingly, two helices from Lamtor1 fill up the gaps on Lamtor4 and Lamtor5, which correspond to the positions of the α3-helices of Lamtor2 and of Lamtor3, thereby stabilizing the Lamtor4-Lamtor5 subcomplex.

A comparison between the Ragulator pentameric complex and the Ego-TC trimeric complex shows that Ego-TC only accounts for half of the Ragulator complex. Ego1, Ego2 and Ego3 are equivalent to the C-terminal half of Lamtor1, Lamtor5 and Lamtor2, respectively. As a rule of thumb, roadblock domain proteins usually exist in pairs, such as the Lamtor2-Lamtor3 pair, the Lamtor4-Lamtor5 pair and the RagA-CTD-RagC-CTD pair. In the roadblock domain protein pairs, their central β-sheets merge together to form a more extensive β-sheet, thus stabilizing each other. However, in the Ego-TC complex, the roadblock domain proteins Ego2 and Ego3 have no contact with each other and apparently exist as two lone roadblock domains. These facts prompt us to propose that there might be additional components of the Ego complex. For example, there might be a roadblock domain protein pairing with Ego2, and another roadblock domain protein pairing with Ego3. Interestingly, it has been suggested previously that Ego3 might function as a homodimer to mediate the interaction between the Gtr1-Gtr2 complex and Ego1 so as to activate the TORC1 complex [18]. Considering the similarity between Ego2 and Ego3, it is quite likely that Ego2 also forms a homodimer. Therefore, in addition to the hypothesis of there existing additional components of the Ego complex, there lies another possibility that Ego1, the Ego2 homodimer and the Ego3 homodimer assemble into a pentameric Ego1-(Ego2)_2_-(Ego3)_2_ complex. Presumably, the N-terminal region of Ego1, which was disordered and could not be observed in the crystal structure of the Ego-TC complex [19], interacts with these additional roadblock domain components of the Ego complex. In this way, Ego1 enwraps Ego2, Ego3 and the additional roadblock domain components together, as Lamtor1 does in the Ragulator complex.

In the mean time when this manuscript was being prepared, a report on the crystal structures of Ragulator and the Ragulator-RagA-CTD-RagC-CTD complex was published by Scheffzek and co-workers [24]. The structural assembly of the Ragulator complex in their report was similar to ours. However, they found that mutation or deletion of the C-terminal L^154^VV^156^ motif of Lamtor1 disrupted the association between Ragulator and the Rag GTPase complex, both by the co-immunoprecipitation and by the immunofluorescence approaches. The discrepancy between our result and theirs might be due to the fact that we used purified recombinant protein to perform the interaction assay *in vitro*, whereas they used *in vivo* methods to examine protein interactions in cells. It is likely that there exist post-translational modifications on components of Ragulator or the Rag GTPase complex besides lipidation of the N terminus of Lamtor1, so that the interaction between Ragulator and the Rag GTPase complex is weakened compared with unmodified proteins as we have used. Certainly, other possibilities might also exist which await further investigations.

## Materials and Methods

### Purification and crystallization of the Lamtor4-Lamtor5 complex

The cDNAs of full-length human Lamtor4 and residues 83–173 of human Lamtor5 were subcloned into a pRSFDuet-1 vector (Novagen, Darmstadt, Germany) with a tobacco etch virus (TEV) protease cleavage site between the 6×His tag and the N terminus of Lamtor5 for recombinant protein expression in the *Escherichia coli* strain BL21(DE3). The Lamtor4-Lamtor5 protein complex was purified using Ni^2+^-NTA affinity chromatography (GE Healthcare, Little Chalfont, UK), and the eluted protein was incubated with the TEV protease (1:50 ratio, w w^−1^) at 4 °C for 12 h to remove the 6×His tag in the presence of β-mercaptoethanol (1:1 000 ratio, v v^−1^). The protein was further purified by anion exchange chromatography, followed by gel filtration chromatography in 10 mM Tris-HCl, pH 8.0, 100 mM NaCl and 2 mM dithiothreitol. The crystals were obtained using the hanging-drop vapor-diffusion method with reservoir solution containing 0.1 m Tris-HCl, pH 8.0, 3.5 m sodium formate at 287 K for 2 days.

### Purification and crystallization of the Ragulator complex

Three two-promoter coexpression vectors were used to coexpress the five subunits of Ragulator complex. Lamtor1 (residues 41–161) was cloned into the multiple cloning sites 1 of pETDuet-1 (Novagen) with a TEV protease cleavage site after the N-terminal 6×His tag. Meanwhile, full-length Lamtor2 and Lamtor3 were subcloned into the two multiple cloning sites of the pACYCDuet-1 vector (Novagen), whereas full-length Lamtor4 and Lamtor5 (residues 83–173) were subcloned into the two multiple cloning sites of the pRSFDuet-1 vector (Novagen). None of these four proteins carry tags. The Ragulator protein complex was expressed in the *Escherichia coli* BL21(DE3) strain. Cells were grown at 37 °C in Luria Broth medium and induced when the optical density at 600 nm became 1.0 by adding 0.3 mM isopropyl thiogalactoside at 23 °C for 12 h. The cultures were harvested by centrifugation and resuspended in 25 mM Tris-HCl, pH 8.0, 20 mM imidazole and 300 mM NaCl. The protein complex was first purified using the Ni^2+^-NTA affinity chromatography, and the eluted protein was incubated with TEV protease (1:50 ratio, w w^−1^) at 4 °C for 12 h to remove the 6×His tag in the presence of β-mercaptoethanol (1:1 000 ratio, v v^−1^). The protein was further purified by anion exchange chromatography, followed by gel filtration chromatography in 10 mM Tris-HCl, pH 8.0, 100 mM NaCl and 2 mM dithiothreitol. Before crystallization, the Ragulator protein complex was concentrated by ultrafiltration to 15 mg ml^−1^. Crystallization was carried out at 287 K by using the hanging-drop vapor-diffusion method. Crystals were grown at 287 K after 1 week by mixing 1 μl of protein with 1 μl of crystallization solution containing 0.1 M CHES (2-[*N*-cyclohexylaminolethanesul-phonic acid), pH 9.5, 0.56 m sodium citrate tribasic and 1.4 m sodium chloride.

For purification of the Ragulator complex assembled with Lamtor1 C-terminal deletion or point mutation mutants, only Lamtor1 (residues 41–161) was replaced by either Lamtor1 (residues 41–150) or Lamtor1 (residues 41–161, L^154^VVQF^158^ mutated to D^154^DDQD^158^), and other procedures remained the same.

### Data collection and structure determination

Crystals were cryoprotected in the crystallization buffer supplemented with 25% glycerol. One set of diffraction data of the Lamtor4-Lamtor5 complex at 2.03 Å resolution and one set of diffraction data of the Ragulator complex at 3.01 Å resolution were collected at the beamline BL19U1 of National Center for Protein Sciences Shanghai at 100 K [25]. Data reduction was performed with the HKL3000 software (Charlottesville, VA, USA) [26]. Phases were determined using the Phaser program [27] in the CCP4 package [28]. Model building was performed using COOT [29], and refinement was performed by the REFMAC5 program [30] in the CCP4 package [28]. The final refined models have *R*_work_/*R*_free_ factors of 21.03%/26.27% and 22.50%/28.54% for the Lamtor4-Lamtor5 and the Ragulator complexes, respectively. The quality of the structure models were checked with the CCP4 program PROCHECK [28], which showed good stereochemistry according to the Ramachandran plot.

### Purification of the RagA-RagC complex

Human RagA (residues 6–302, with the point mutation T21N) and human RagC (residues 60–375, with the point mutation Q120L) were subcloned into the pETDuet-1 vector (Novagen) for recombinant protein expression in the *Escherichia coli* strain BL21(DE3). RagA carries an N-terminal 6×His tag, whereas RagC has no tag. Bacteria cells were incubated at 37 °C until the optical density (OD) at 600 nm reached 1.0. Protein expression was induced with 0.2 mM isopropyl thiogalactoside and the cells were further incubated at 16 °C for 16–20 h. The cultures were harvested by centrifugation and resuspended using 25 mM Tris-HCl, pH 8.0, 20 mM imidazole and 300 mM NaCl. After lysis of bacterial cells with a cell homogenizer (JNBIO, Guangzhou, China) and centrifugation, the RagA-RagC protein complex was purified using Ni^2+^-NTA affinity chromatography and anion exchange chromatography, followed by gel filtration chromatography in 15 mM Tris-HCl, pH 8.0, 150 mM NaCl and 4 mM dithiothreitol. The same procedure was used to purify the RagA-CTD (residues 183–302)-RagC-CTD (residues 239–375) protein complex.

### Ni^2+^-NTA affinity chromatography assay between the Ragulator complex and the RagA-RagC GTPase complex

Ni^2+^-NTA affinity chromatography pull-down assay was performed using purified 6×His-tagged RagA (residues 6–302, T21N)-RagC (residues 60–375, Q120L) complex and untagged Ragulator complex, with the 6×His tag on Lamtor1 already removed by the TEV protease. The 6×His-tagged RagA (6–302, T21N)-RagC (60–375, Q120L) protein complex was diluted to 1 mg ml^−1^. The Ragulator complex was diluted to 0.5 mg ml^−1^. 6×His-tagged RagA (6–302, T21N)-RagC (60–375, Q120L) was first loaded onto the Ni^2+^-NTA affinity column. The flow-through was collected and the Ni^2+^-NTA affinity column was washed with 20 column volumes of equilibrium buffer (25 mM Tris-HCl, pH 8.0, 300 mM NaCl and 20 mM imidazole). Untagged Ragulator was then loaded onto the Ni^2+^-NTA column and the flow-through was collected. The column was washed with another 20 column volumes of equilibrium buffer, and then eluted with 3 column volumes of the elution buffer (25 mM Tris-HCl, pH 8.0, 300 mM NaCl and 300 mM imidazole). Ni^2+^-NTA affinity chromatography pull-down assay between 6×His-tagged RagA-CTD (residues 183–302)−RagC-CTD (residues 239–375) complex and untagged Ragulator complex was performed using the same method.

### Molecular graphics

All the protein structure figures were generated by the PyMOL program [31].

### Accession codes

The structural coordinates and structural factors have been deposited into the Protein data Bank (PDB) with codes of 5YK3 and 5YK5 for human Ragulator complex and human Lamtor4-Lamtor5 complex, respectively.

## Figures and Tables

**Table 1:** Data collection and refinement statistics

	*Lamtor4-Lamtor5*	*The Ragulator complex*
*Data collection*		
Beamline	BL19U1	BL19U1
Space group	*P*3_2_	*P*6_1_22
Cell dimensions		
*a*, *b*, *c* (Å)	76.67, 76.67, 52.94	126.68, 126.68, 614.51
*α*, *β*, *γ* (deg.)	90, 90, 120	90, 90, 120
Resolution (Å)	50–2.03 (2.10–2.03)	50–3.10 (3.21–3.10)
*R*_merge_ (%)	9.0 (>100.0)	20.1 (>100.0)
*I*/*σ_I_*	20.5 (1.75)	28.5 (2.25)
CC_1/2_	0.548	0.797
Completeness (%)	100.0 (100.0)	100.0 (100.0)
Redundancy	10.3 (9.2)	34.6 (20.3)
Wilson-plot *B* factor	24.5	52.1
Molecules/asymmetric unit	2 complexes	3 complexes
		
*Refinement*		
Resolution (Å)	50–2.03	50–3.10
Number of reflections	19 847	47 579
*R*_work_/*R*_free_	21.64%/26.24%	22.27%/28.33%
No. of atoms		
Protein	2 542	11 608
Solvent	68	0
*B* factors (Å^2^)		
Overall	13.53	58.51
Protein	13.13	58.51
Solvent	28.53	N/A
RMSD bond length (Å)	0.0070	0.0056
RMSD bond angles (deg.)	1.4407	1.2122
Ramachandran plot		
Favored (%)	93.4	94.8
Allowed (%)	6.3	4.9
Disallowed (%)	0.3	0.3

Abbreviations: ASU, asymmetric unit; N/A, not applicable; RMSD, root-mean-square deviations from ideal geometry.

*R*_merge_=Σ*_h_*Σ*_i_* |*I_h,i_*−*I_h_*|/Σ*_h_*Σ*_i_* *I_h,i_* for the intensity (*I*) of observation *i* of reflection *h*. *R* factor=Σ||*F*_obs_|−|*F*_calc_||/Σ|*F*_obs_|, where *F*_obs_ and *F*_calc_ are the observed and calculated structure factors, respectively. *R*_free_=*R* factor calculated using 5% of the reflection data chosen randomly and omitted from the start of refinement. Data for the highest resolution shell are shown within parentheses.
