# Supplementary material for: Structural insight into the Ragulator complex which anchors mTORC1 to the lysosomal membrane
Source: Cell Discov. 2017 Dec 26;3:17049–. doi: 10.1038/celldisc.2017.49 (PMC5742854; doi:10.1038/celldisc.2017.49)
Supplement: Supplementary Information [file celldisc201749-s1.pdf]

Supplementary information

Supplementary Figures

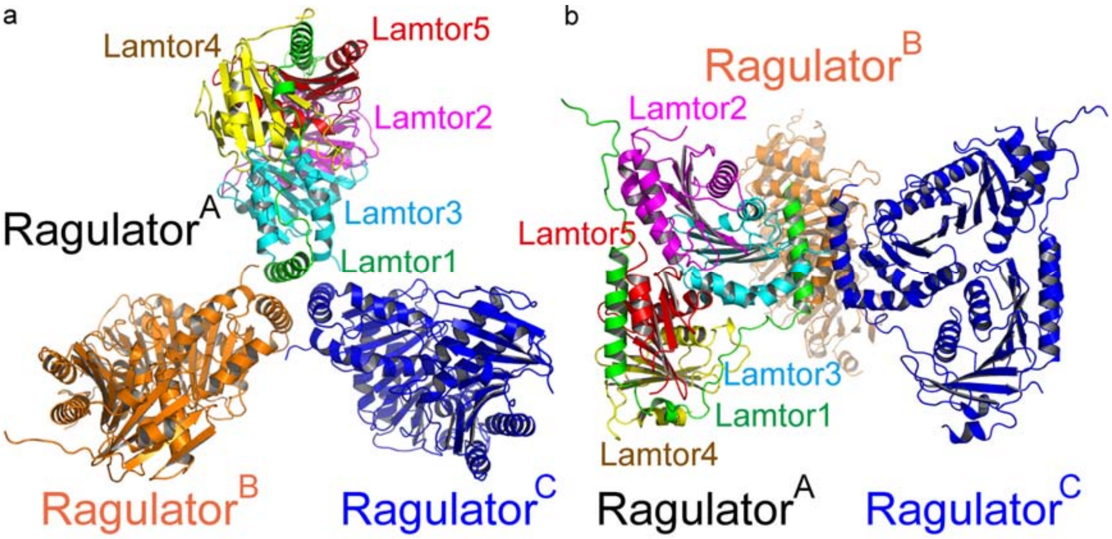

Supplementary Figure S1 The trimeric assembly of Ragulator in the asymmetric unit of the crystal structure. The three Ragulator complexes in the asymmetric unit are denoted A, B, and C, respectively. The Lamtor1, Lamtor2, Lamtor3, Lamtor4, and Lamtor5 subunits of Ragulator^{A} are colored in green, magenta, cyan, yellow, and red, respectively. Ragulator^{B} and Ragulator^{C} are colored in orange and blue, respectively. **a**, Top view. **b**, Side view.

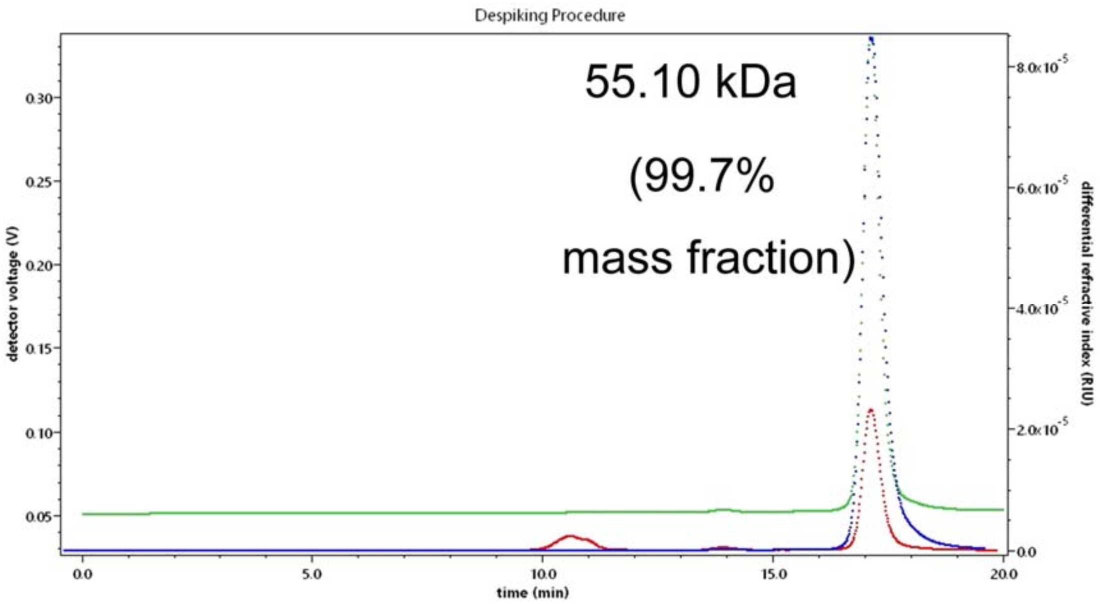

Supplementary Figure S2 The Ragulator complex is monomeric in solution. The molecular weight of the Ragulator complex was measured to be 55.10 kDa by the size exclusion chromatography − multi-angle light scattering (SEC-MALS) method. The green, blue, and red profiles represent Ultra-Violet absorption, differential refractive index, and light scattering measurements, respectively.

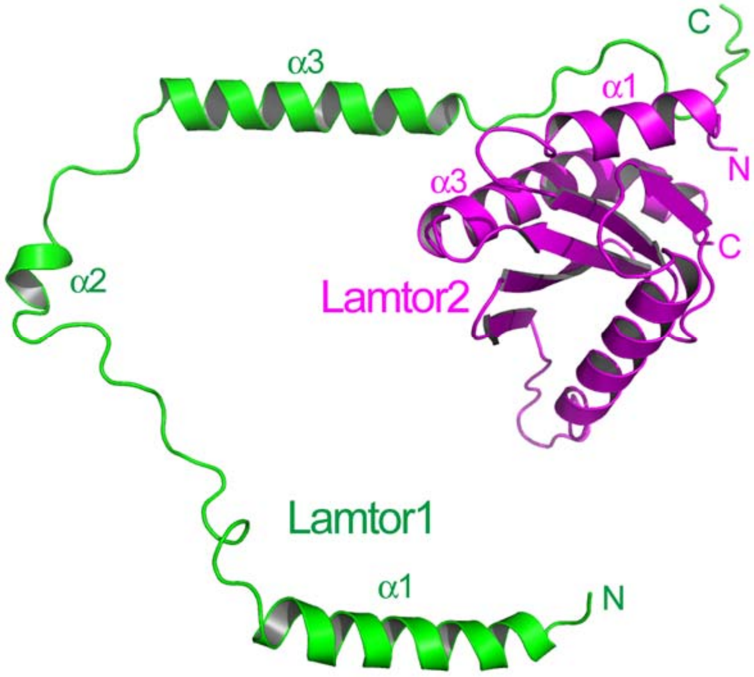

Supplementary Figure S3 Overall structure of the Lamtor1-Lamtor2 sub-complex of the Ragulator complex. The extended C-terminus of Lamtor1 interacts with the α1 and α3 helices of Lamtor2. Lamtor1 and Lamtor2 are colored in green and magenta, respectively.

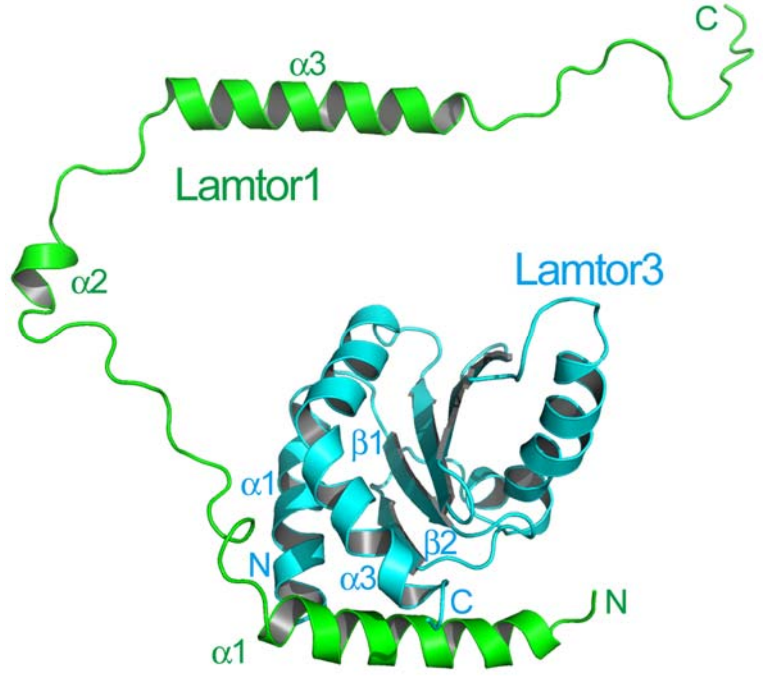

Supplementary Figure S4 Overall structure of the Lamtor1-Lamtor3 sub-complex of the Ragulator complex. The α1 helix of Lamtor1 interacts with the α1 and α3 helices, as well as the β1-β2 loop of Lamtor3. Lamtor1 and Lamtor3 are colored in green and cyan, respectively.

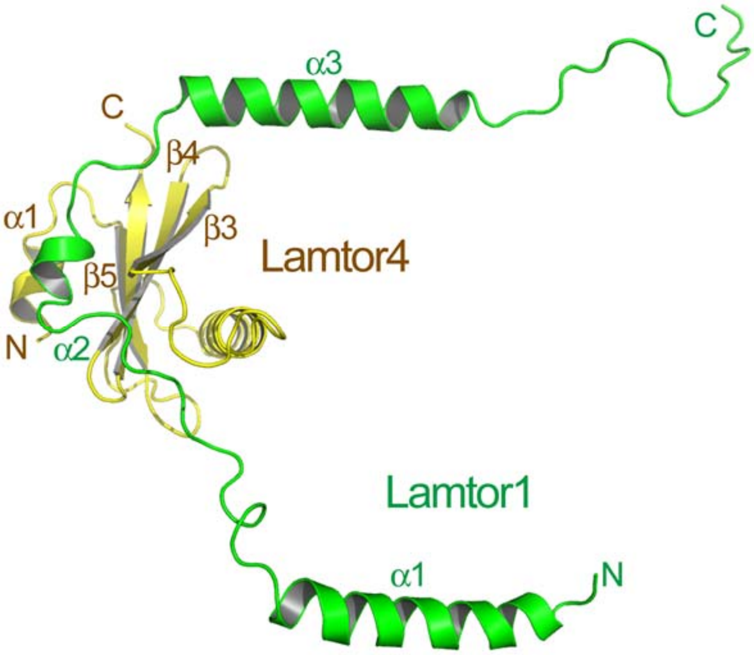

Supplementary Figure S5 Overall structure of the Lamtor1-Lamtor4 sub-complex of the Ragulator complex. The α2 helix of Lamtor1 interacts with the α1 helix, as well as the β3, β4, and β5 strands of Lamtor4. Lamtor1 and Lamtor4 are colored in green and yellow, respectively.

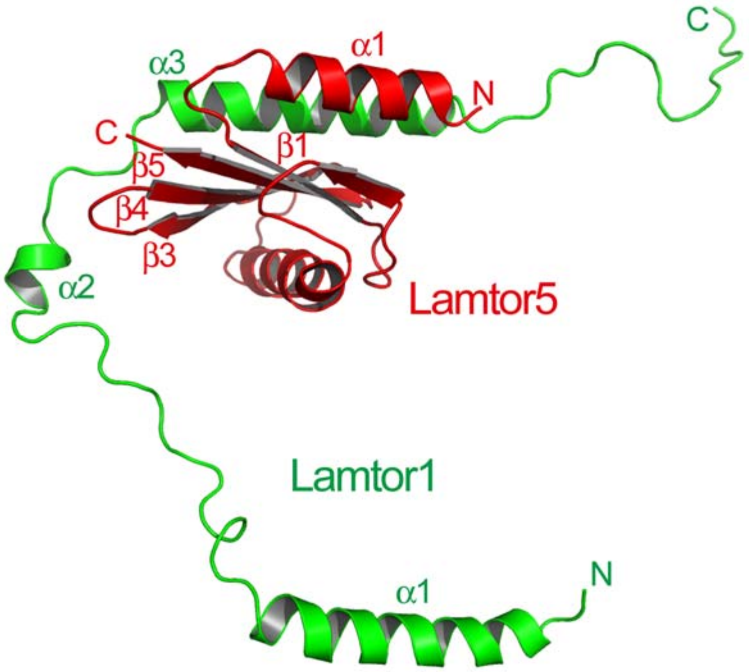

Supplementary Figure S6 Overall structure of the Lamtor1-Lamtor5 sub-complex of the Ragulator complex. The α3 helix of Lamtor1 interacts with the α1 helix, as well as the β1, β3, β4, and β5 strands of Lamtor5. Lamtor1 and Lamtor5 are colored in green and red, respectively.

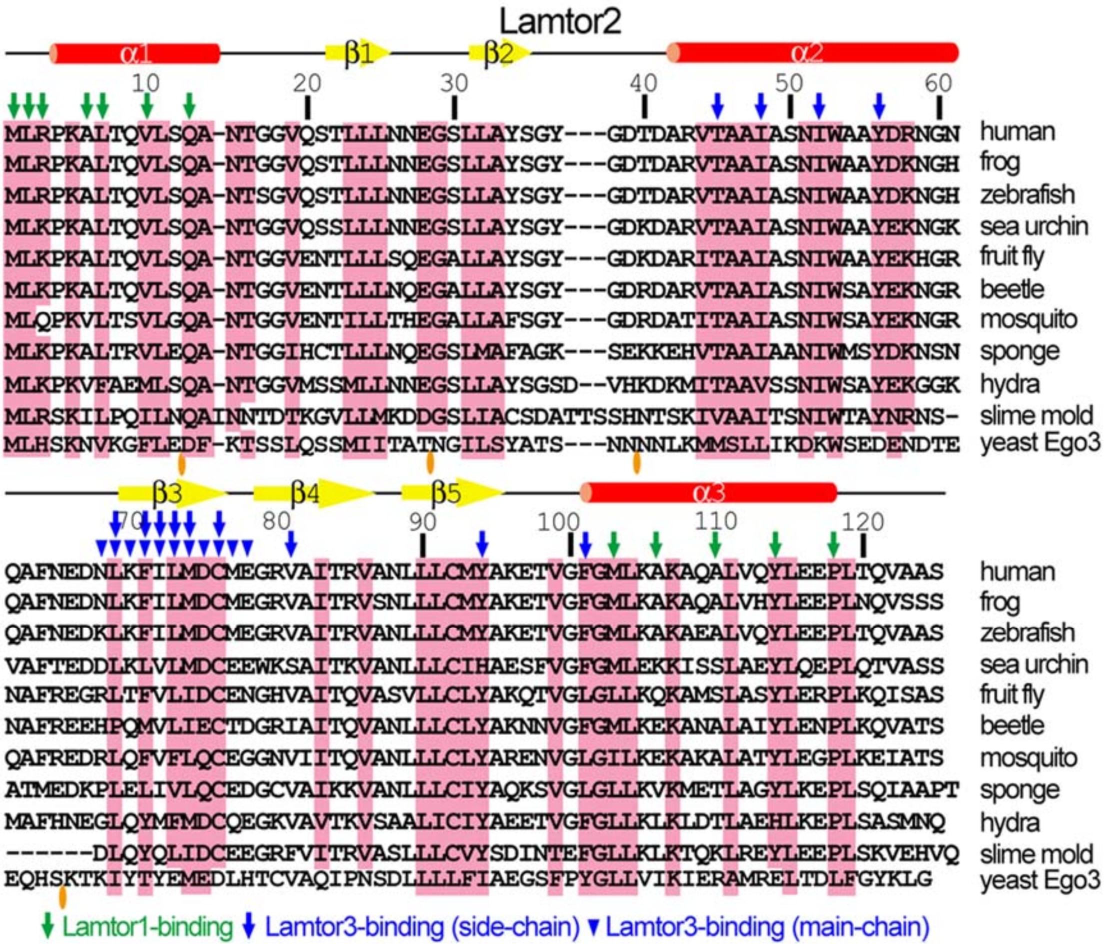

Supplementary Figure S7 Structure-based multiple sequence alignment of Lamtor2 (also knows as p14) orthologues from various species. α helices and β sheets are shown as red cylinders and yellow arrows, respectively. Every ten residue of human Lamtor2 is marked. Conserved residues are shaded in pink. Residues mediating interactions with Lamtor1, Lamtor3 (by side-chains), and Lamtor3 (by main-chains), are highlighted by green arrows, blue arrows, and blue arrowheads, respectively. The places which contain loop-insertions are indicated with orange ovals.

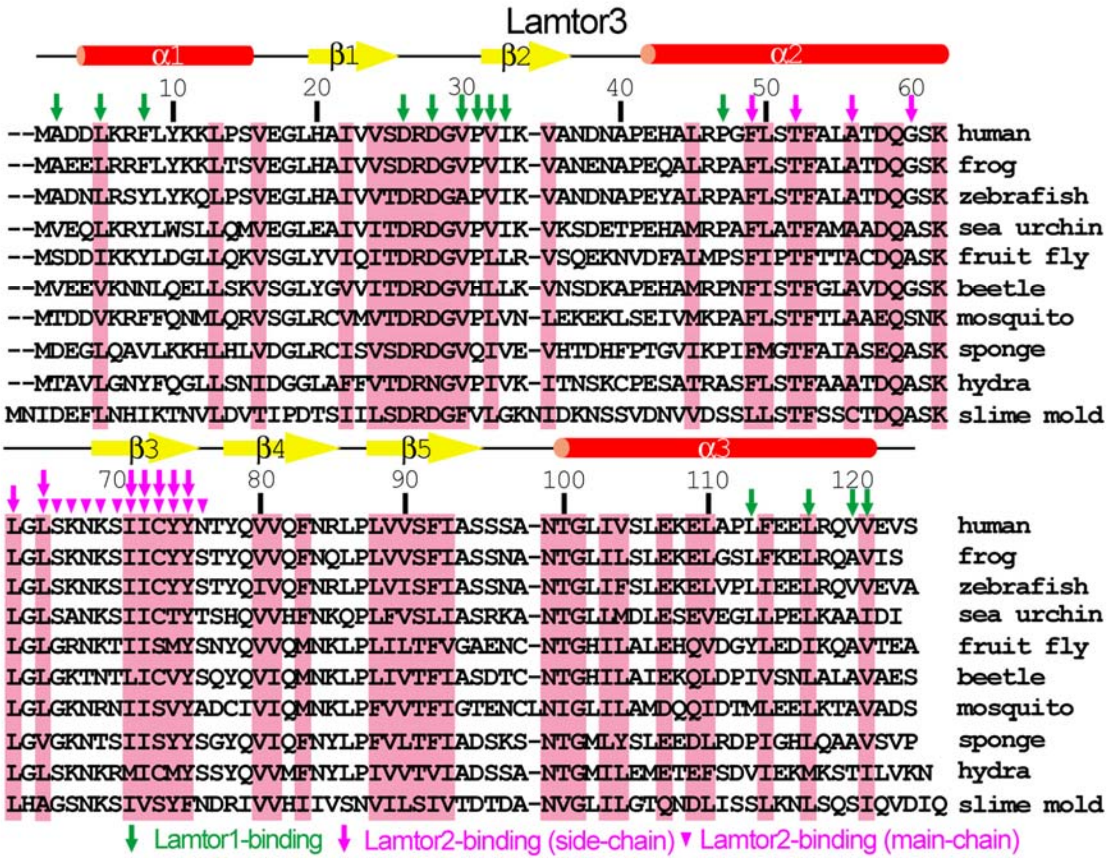

Supplementary Figure S8 Structure-based multiple sequence alignment of Lamtor3 (also knows as MP1) orthologues from various species. α helices and β sheets are shown as red cylinders and yellow arrows, respectively. Every ten residue of human Lamtor3 is marked. Conserved residues are shaded in pink. Residues mediating interactions with Lamtor1, Lamtor2 (by side-chains), and Lamtor2 (by main-chains) are indicated by green arrows, magenta arrows, and magenta arrowheads, respectively.

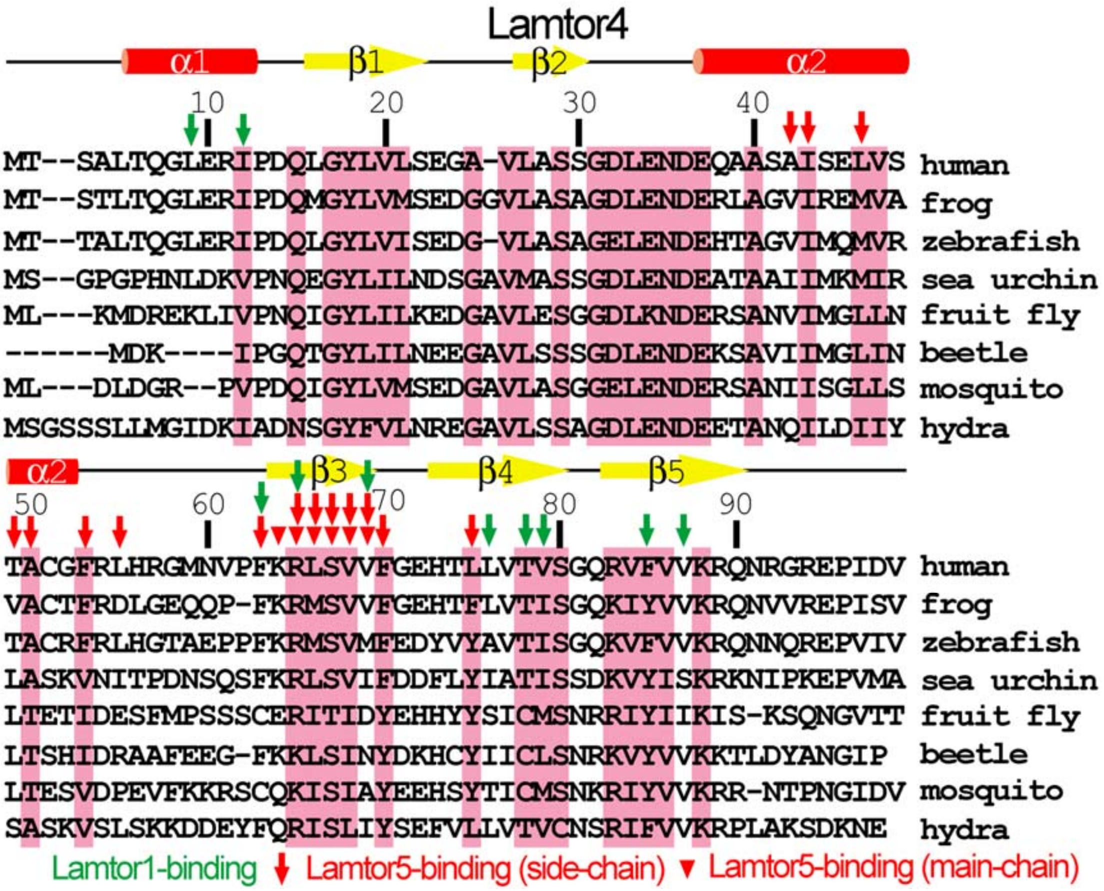

Supplementary Figure S9 Structure-based multiple sequence alignment of Lamtor4 orthologues from various species. α helices and β sheets are shown as red cylinders and yellow arrows, respectively. Every ten residue of human Lamtor4 is marked. Conserved residues are shaded in pink. Residues mediating interactions with Lamtor1, Lamtor5 (by side-chains), and Lamtor5 (by main-chains) are indicated by green arrows, red arrows, and red arrowheads, respectively.

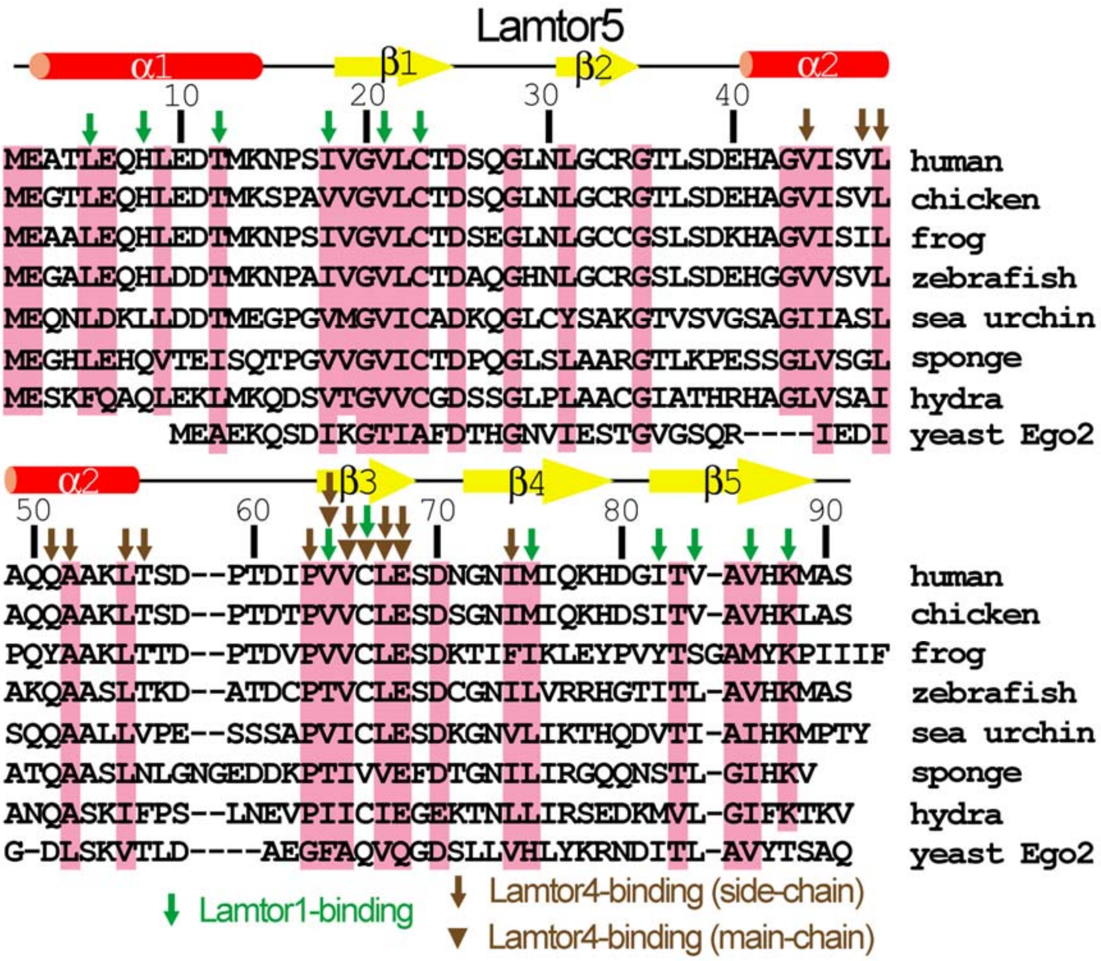

Supplementary Figure S10 Structure-based multiple sequence alignment of Lamtor5 orthologues from various species. α helices and β sheets are shown as red cylinders and yellow arrows, respectively. Every ten residue of human Lamtor5 is marked. Conserved residues are shaded in pink. Residues mediating interactions with Lamtor1, Lamtor2, Lamtor4 (by side-chains), and Lamtor4 (by main-chains) are indicated by green arrows, magenta arrows, brown arrows, and brown arrowheads, respectively.

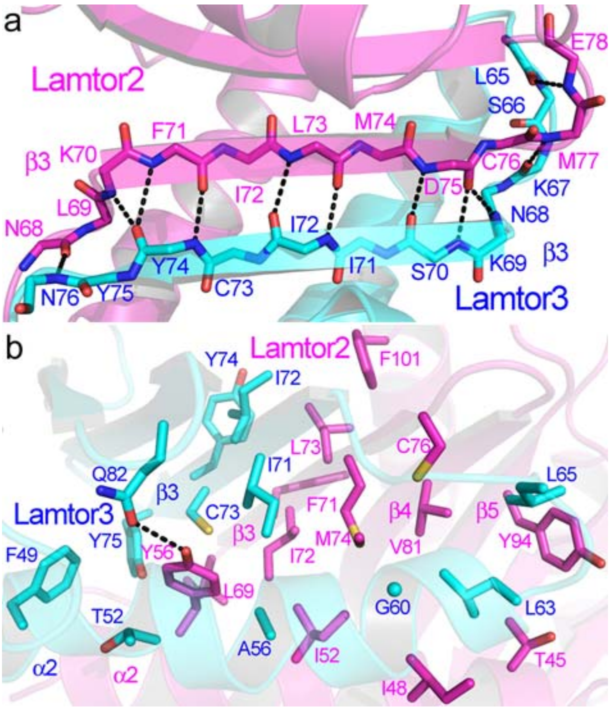

Supplementary Figure S11 The interaction interface between Lamtor2 and Lamtor3. a, β strands pairing between main-chains of Lamtor2 and Lamtor3 residues. **b**, van der Waals interactions and hydrogen bonds between side-chains of Lamtor2 and Lamtor3 residues. Carbon atoms of Lamtor2 and Lamtor2 are colored in magenta and cyan, respectively. Nitrogen and oxygen atoms are colored in blue and red, respectively. Hydrogen bonds are shown as black dashed lines.

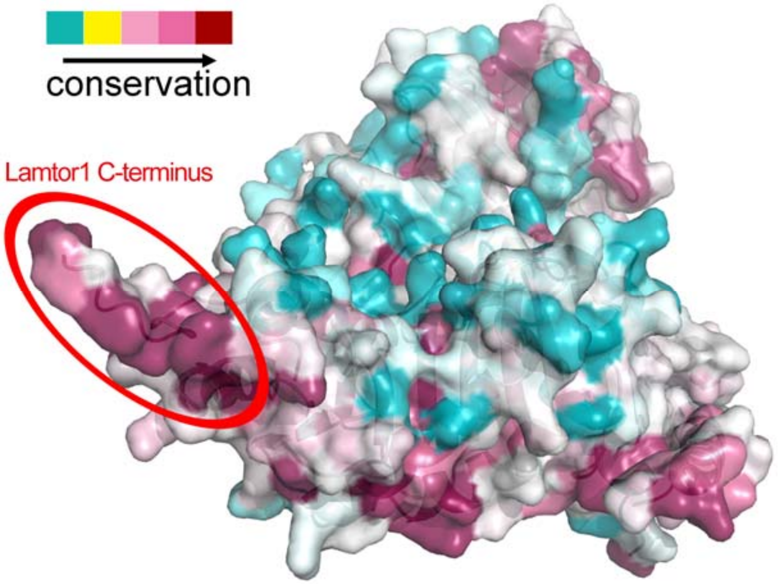

Supplementary Figure S12 The C-terminal region of Lamtor1 is among the most conserved parts of the Ragulator surface. The sequence conservation of every subunit of the Ragulator complex is scored and mapped onto the surface of its structure, with red and cyan representing the highest and the lowest conservation scores, respectively.

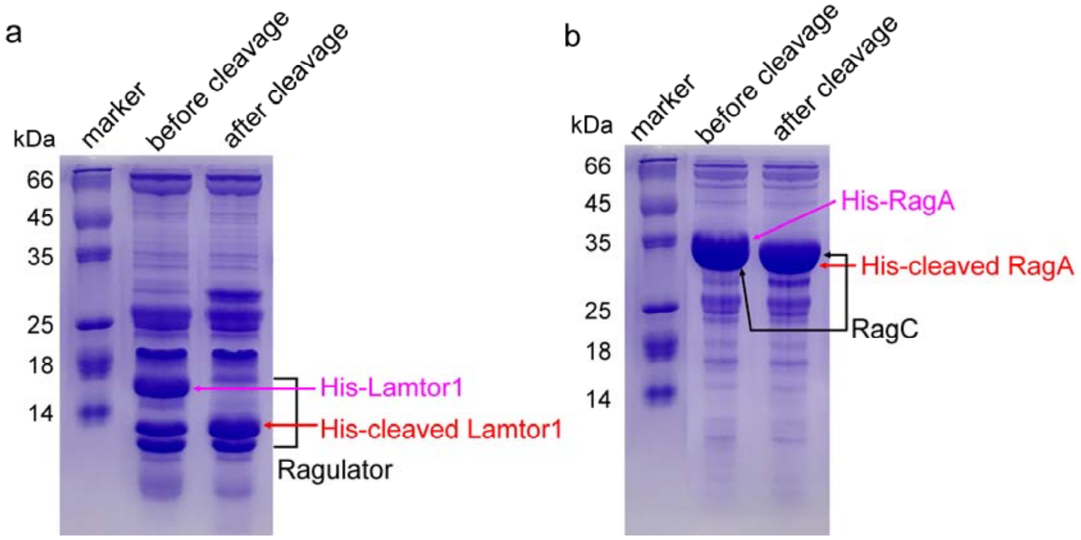

Supplementary Figure S13 The 6×His tag on Lamtor1 (41-161) in the Ragulator complex and the 6×His tag on RagA in the RagA-RagC complex were indeed cleaved off after the cleavage reaction. **a**, After the cleavage reaction, the 6×His tag on Lamtor1 (41-161) in the Ragulator complex was indeed cleaved off. **b**, After the cleavage reaction, the 6×His tag on RagA in the RagA-RagC complex was indeed cleaved off.
